# On Glycosuria as an Accompaniment of Marsh Fevers

**Published:** 1861-03

**Authors:** 


### ON GLYCOSURIA AS AN ACCOMPANIMENT OF MARSH FEVERS.

BY DR. BURDEL, Physician to the Vierzon Hospital.

Dr. Burdel regards marsh poison as a myth, and looks upon marsh fever as a result of a perturbation of the cerebro spinal centre and the sympathetic system, adopting very nearly the same phrase as the one by which Bernard defines glycosuria. The author of the present paper, in his researches into the nature of marsh fever, has confirmed the above view of its character by ascertaining in the majority of cases the presence of sugar in the urine.

Dr. Burdel employed the test with liquor potassæ, Feeling's liquids, the test with bismuth and potash or carbonate of soda, and the yeast test. It was especially in the first commencement of the attack that the quantity of sugar was considerable; it diminished gradually towards the termination of the paroxysm, and generally disappeared entirely during the interval. The closer the attacks approach one another, the larger the amount of sugar.

In eighty cases of well marked intermittent fever, the author uniformly found sugar; in thirty other cases, in which the fever was at first intermittent and subsequently became remittent, the sugar was present, but only in small quantity and for brief space. In two cases of intermittent fever, following typhoid fever, a considerable quantity of sugar was shown to be present.

In the case presenting the largest quantity of sugar, as much as 10 per 1000 was found.—*L'Union Médicale.*

